# Traumatic brain injury neuroelectrochemical monitoring: behind-the-ear micro-instrument and cloud application

**DOI:** 10.1186/s12984-020-00742-x

**Published:** 2020-08-21

**Authors:** Momen K. Tageldeen, Sally A. N. Gowers, Chi L. Leong, Martyn G. Boutelle, Emmanuel M. Drakakis

**Affiliations:** 1 Bioinspired VLSI Circuits and Systems Group, Department of Bioengineering, Imperial College London, Exhibition Road, London, SW7 2AZ UK; 2 Biomedical Sensors Group, Department of Bioengineering, Imperial College London, Exhibition Road, London, SW7 2AZ UK

**Keywords:** Traumatic brain injury (TBI), Flexible PCB, Wireless brain monitoring, Cloud processing, Microdialysis

## Abstract

**Background:**

Traumatic Brain Injury (TBI) is a leading cause of fatality and disability worldwide, partly due to the occurrence of secondary injury and late interventions. Correct diagnosis and timely monitoring ensure effective medical intervention aimed at improving clinical outcome. However, due to the limitations in size and cost of current ambulatory bioinstruments, they cannot be used to monitor patients who may still be at risk of secondary injury outside the ICU.

**Methods:**

We propose a complete system consisting of a wearable wireless bioinstrument and a cloud-based application for real-time TBI monitoring. The bioinstrument can simultaneously record up to ten channels including both ECoG biopotential and neurochemicals (e.g. potassium, glucose and lactate), and supports various electrochemical methods including potentiometry, amperometry and cyclic voltammetry. All channels support variable gain programming to automatically tune the input dynamic range and address biosensors’ falling sensitivity. The instrument is flexible and can be folded to occupy a small space behind the ear. A Bluetooth Low-Energy (BLE) receiver is used to wirelessly connect the instrument to a cloud application where the recorded data is stored, processed and visualised in real-time. Bench testing has been used to validate device performance.

**Results:**

The instrument successfully monitored spreading depolarisations (SDs) - reproduced using a signal generator - with an SNR of 29.07 dB and NF of 0.26 dB. The potentiostat generates a wide voltage range from -1.65V to +1.65V with a resolution of 0.8mV and the sensitivity of the amperometric AFE was verified by recording 5 pA currents. Different potassium, glucose and lactate concentrations prepared in lab were accurately measured and their respective working curves were constructed. Finally,the instrument achieved a maximum sampling rate of 1.25 ksps/channel with a throughput of 105 kbps. All measurements were successfully received at the cloud.

**Conclusion:**

The proposed instrument uniquely positions itself by presenting an aggressive optimisation of size and cost while maintaining high measurement accuracy. The system can effectively extend neuroelectrochemical monitoring to all TBI patients including those who are mobile and those who are outside the ICU. Finally, data recorded in the cloud application could be used to help diagnosis and guide rehabilitation.

## Background

Traumatic brain injury (TBI) is a non-degenerative, non-congenital condition; it could be defined as a set of perceptible and non-perceptible brain insults due to an external impact on the head. Such insults include brain herniation, haemorrhage and contusion [1, 2]. In addition to the primary injury that occurs at the moment of impact, secondary injuries are likely to develop especially in mild and severe TBI injuries [3]. These secondary injuries may take hours or even days to manifest and can lead to reduction in life expectancy, altered level of consciousness, post-traumatic disorder and neurological disorders, along with other cognitive and psychological/psychiatric impairments [3]. Hence, TBI should not be viewed as a single event but rather as a sustained condition calling for monitoring, supervision and rehabilitation [4].

TBI is characterised by a complex pathway where patients could relapse after surgery, while in the intensive care unit (ICU), the high dependency unit (HDU) or in normal hospital wards, and may require additional invasive interventions. The motivation for neuroelectrochemical monitoring of TBI patients stems from the need for timely neurological intervention and prevention of adverse effects. However, currently, TBI neuroelectrochemical monitoring is limited to fully sedated patients in the ICU who have undergone craniotomy neurosurgery as a result of sustaining a severe TBI and/or showing acute brain insults [5, 6].

A closely monitored event in TBI is the onset of spreading depolarisations (SDs), also referred to as “brain tsunamis”. These are slow changing mass depolarization waves that originate from the lesion foci and spread out to neighbouring tissues at risk of secondary injury [7]. SDs are strongly associated with poor outcome in TBI patients and are measured by means of electrocorticography (ECoG). Subdural strip electrodes are traditionally used to monitor SD events, however, they require the patient to undergo craniotomy. Recently, intraparenchymal electrodes were used to accurately monitor these events; these electrodes are inserted via burr hole which is a minimally-invasive procedure [8].

Chemical monitoring of the injured brain can give an indication of tissue health and metabolism [9]. In 2014, a consensus statement from the International Microdialysis Collaborative Group identified glucose and the lactate/pyruvate (L/P) ratio as the most relevant neurochemical biomarkers in TBI monitoring [10]. Glucose concentration reflects local metabolism, hence, poor outcomes have a direct link to low glucose concentrations (< 0.8 mM at 0.3 l/min in tissue interstitial space) [11–15]. Likewise, the association of abnormally high glucose concentrations with poor outcome has also been reported [16, 17] reflecting failure of glucose metabolism due to local tissue death. Absolute concentrations of lactate and pyruvate, in conjunction with the L/P ratio provide information about the cellular redox state in the area of interest. Relatively high concentrations of lactate can be due to both ischemic and non-inshemic (e.g. mitochondrial dysfunction) causes [18, 19]. Continuous on-line microdialysis (co-MD) is used for sampling brain extracellular fluid to measure neurochemical biomarker concentrations and changes [6]. A single microdialysis probe perfused with a physiological solution is inserted into the monitored region either during craniotomy or through cranial bolt in such a way as to cause minimum tissue disruption. Intraparenchymal ECoG electrodes can be inserted through the same bolt as the microdialysis probe which leads to minimally-invasive monitoring [8]. Potassium measurement should also be included in order to enable a spatiotemporal correlation between the chemical and electrical measurements: potassium measurements can chemically denote the onset of SDs [20].

**Fig. 1:**
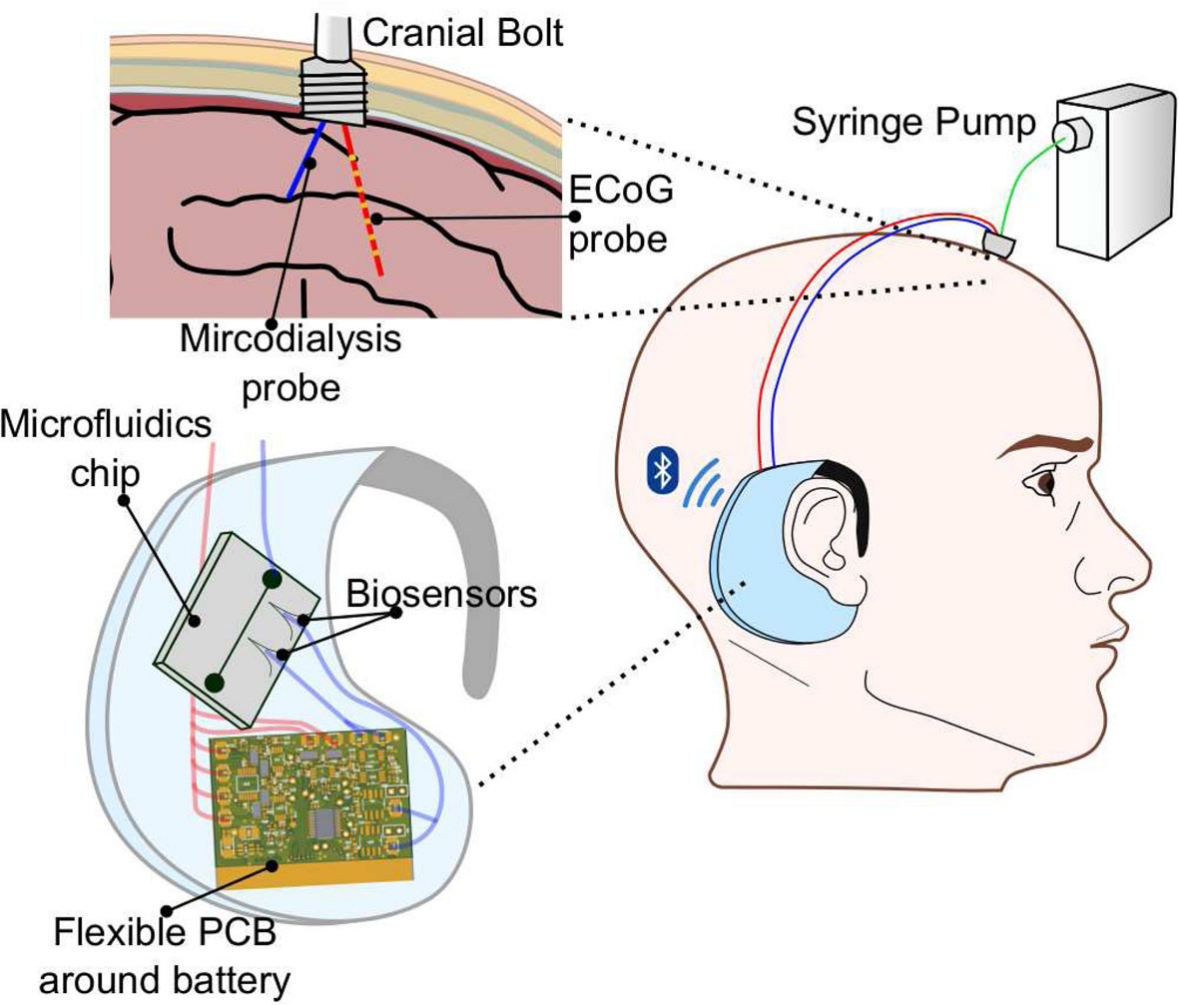
The setup of the proposed behind-the-ear wearable device. The solution consists of: the micro-instrument (flexible PCB), microfluidic chip and biosensors. The device connects to a minimally-invasive cranial bolt, which is fixed on the patient’s head where the injury is. The bolt has two lumens, one for an ECoG probe and the other for the microdialysis probe. The latter requires a syringe pump to perfuse the probe membrane. The device is wireless and supports bluetooth low-energy (BLE) protocol

In summary, the device has to measure the biopotential ECoG signals with a resolution sufficient to identify the SDs and make inference on the depolarization of the injured brain [6]. It also has to support amperometry to measure glucose and lactate, as well as potentiometry to measure potassium. This article presents a complete system for monitoring TBI that consists of a wearable bioinstrument (Fig. 1) and a cloud application for data visualisation and analysis.

The lack of an affordable, wearable, relatively non-invasive instrument is the major barrier for real-time neuroelectrochemical monitoring of patients that are mobile and in risk of secondary injuries; or patients in low-income countries, military and “curb-medicine” settings. Several studies have reported instruments for brain monitoring. An integrated chip for wireless neurochemical measurement was proposed by Roham et al. (2008) [21]. The chip offered both amperometry and fast-scan cyclic voltammetry, However, it had a limited number of channels and a reduced resolution at high sampling rate. In the same vein, Kasasbeh et al. (2013) presented a device that is also limited to neurochemical measurements. Additionally, it is not wearable and is instead designed to be attached to neurosurgical stereotactic frames [22]. In contrast, Piangerelli et al. (2014) proposed an invasive instrument restricted to cortical/electrical signals [23]. Other studies in the literature focused on the design of probes aimed at improving the usability and wearablity of TBI instruments [24].

Pivoting to instruments particularly designed for TBI or neuroelectrochemical monitoring, Papadimitriou et al. (2016) developed a high-performance two board solution. The boards, one for biopotential recording and the other for chemical biomarker measurement, were designed to realise an ambulatory bedside equipment, thus it employed a wired connection to a data collection system and occupied an area of around 400 cm^2^ per board [5]. In Zafeiropoulos, Papadimitriou et al. (2018), PANACEA, an integrated instrument for cortical and neurochemical signals was developed. Most notably, the instrument could be connected to a receiver either through a wired connection or wirelessly via IEEE 802.15.4 (Zigbee) protocol. The latter employing an external antenna had a sampling rate up to 1 ksps/channel and traded power for performance [25]. Again, the instrument, though portable, was not designed as a wearable solution and remained somewhat too large (around 80 cm^2^) to be wearable.

In comparison to ambulatory bed-side instruments, a wearable instrument would allow shorter connection tubes between the patient and the analysis system. This would also facilitate real-time monitoring and enhance temporal resolution because sampled chemical biomarkers would reach the instrument faster. Pagkalos’s “LENBIC” (Low-power Electrical and Neurochemical Biosensor Interfacing Chip) is a high-performance application specific integrated circuit for TBI [26]. LENBIC offers dramatic footprint reduction (7.5 mm^2^), high accuracy and ultra-low power performance. However, the chip - being an analog front-end (AFE) - requires additional peripherals, such as embedded controller and integrated wireless transceivers, which increase the device footprint and cost.

**Fig. 2:**
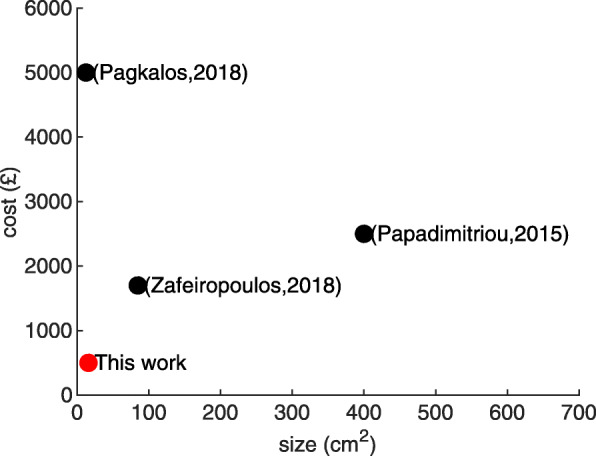
Position of this work in literature. This work presents an aggressive optimisation for size and cost in comparison with other relevant work in the literature. This has been achieved by sacrificing a degree of performance and by precise tailoring of the design to the measured signal properties

What we present here is a wearable micro-instrument specific for full neuroelectrochemical TBI monitoring that trades off performance for an optimal balance between size and cost. This is done while keeping the performance high enough to accurately and sufficiently monitor TBI related biomarkers and events. This trade-off is depicted in Fig. 2 with respect to the other instruments described above. In this article, the performance of the prototype has been validated using bench testing.

## Methods

In this section, we describe a holistic implementation that not only comprises hardware capabilities for accurate TBI neuroelectrochemical monitoring, but also includes a software infrastructure that enables the storage, analysis, and visualisation of real-time measurements.

### Hardware architecture

**Fig. 3:**
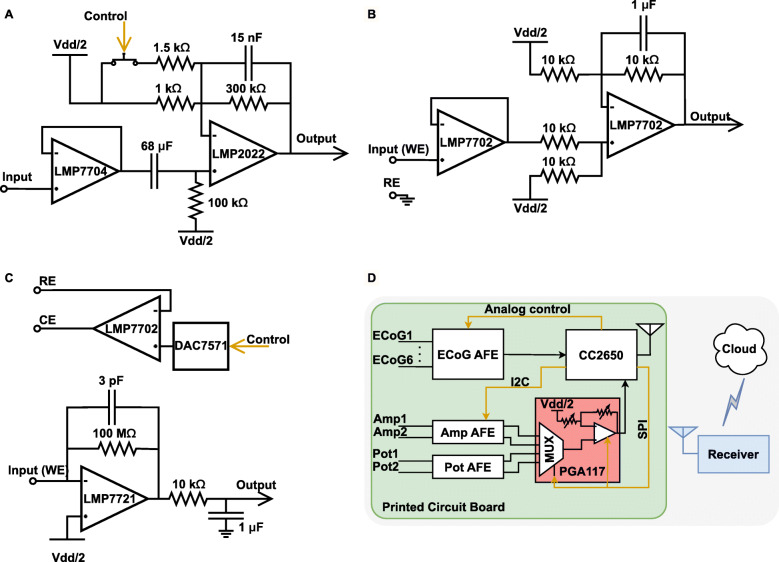
Circuit schematics and device integration. The design and schematics of : **a** The ECoG AFE with controllable gain values of × 300 and × 500, and quasi-DC to 30 Hz bandwidth. **b** The potentiometric (Pot) AFE with unity gain and bandwidth of 10 Hz. **c** The amperometric (Amp) AFE consisting of a 100 M *Ω* transimpedance amplifier (100 mV/nA gain) and 12-bit potentiostat. **d** The complete system overview presenting the integration of the different AFEs and the PGA stage (shown in red). The signal pathway from the inputs, through the PCB, to the receiver and finally to the cloud is also shown

Figure 3 depicts the schematics and integration of the blocks making up the instrument. The ECoG analog front-end (AFE) shown in Fig. 3a can interface with six sub-dural and intraparenchymal electrodes [6, 8]. This allows flexible monitoring of SDs in an injured human brain. This AFE buffers and amplifies bipolar biopotentials with a selectable gain value of × 300 or × 500. These gain options allow optimisation of the system dynamic range under different input signal amplitudes for full exploitation of the analog-to-digital converter (ADC) resolution. The gain switching for the ECoG AFE is achieved by controlling an analog switch (Texas Instruments, TS5A21366) that adjusts the value of the input resistance. The bandwidth of interest in ECoG/SD signals lies between quasi-DC and up to 30 Hz [5]. Hence, analog filters are implemented around the amplifier for anti-aliasing and noise removal. Additionally, the data has been digitally filtered on the server side via a 17th-order Chebyshev II low-pass filter with a stopband edge at 30Hz. The filter coefficients have been generated using MATLAB and the digital filter was implemented using Python on the server side.

The design for both the potentiometric and amperometric AFEs is centred around the fact that neurochemical signals (glucose, lactate, potassium) vary slowly, and thus are characterised by a bandwidth of less than 10 Hz. This feature is exploited as follows: instead of having a dedicated amplification gain stage for each channel, all the chemical channels are multiplexed into one programmable gain amplifier (PGA) minimising the area profile. Figure 3b illustrates the bipolar potentiometric AFE. This stage is dual-channel and accepts voltage signals from ion selective electrodes such as potassium. Here, the AFE functions as a filter and a voltage level shifter with unity gain. Additional gain can be applied at the PGA stage.

The amperometric AFE, shown in Fig. 3c, has two main functions: to deliver a precise potential difference between the biosensor working (WE) and reference (RE) electrodes, and to convert small sensor-induced, pA-range currents to voltage signals. The amperometric AFE comprises two channels, with each channel having a dedicated potentiostat that can generate a voltage difference between -1.65 V to 1.65 V with 12-bit resolution (i.e. 0.8 mV). The potentiostat enables setting and adjusting of the reaction potential, this provides flexibility as the potential can be adjusted depending on the analyte to be detected. Also, it can run sensor calibrations using a variety of electrochemical methods such as cyclic voltammetry, square-wave voltammerty, etc. Finally, the current-to-voltage (I-V) conversion function of the AFE is achieved by a transimpedance amplifier with a gain of 100 mV/nA and a bandwidth of 10 Hz. This AFE is dual-channel and accepts bipolar signals (i.e. sinking and sourcing current). Admittedly, switched capacitor I-V stages are less noisy than feedback-resistor-based transimpedance amplifiers (TIA) [27]. However, switched capacitor approaches lead to increased footprints as they require more components and additional control signals. Also, they are more suitable for high-cost silicon implementation rather than lower-cost printed circuit boards (PCB). In our approach, instead of using one large resistor, the gain is divided between the AFE and the PGA stage.

The PGA stage is based on Texas Instrument’s PG117 and can provide gain values of × 1,×2,×5,×10,×50,×100 and × 200. The PGA117 also includes a builtin multiplexer. As previously mentioned, The low dynamics of the chemical signals are exploited by multiplexing all the chemical channels to one programmable gain stage. This saves area on the board and increases the efficiency of data transfer later on. Figure 3d illustrates the functional integration of the AFEs and the PGA. This figure also shows the organisation of the system including the microcontroller (CC2650) and the cloud connection. CC2650 was chosen because of its compact size and its built-in Bluetooth Low Energy (BLE) transceiver. While BLE, Adaptive Network Topology (ANT), and Zigbee are the most commonly used wireless communication protocols for low-power sensor networks and the internet of things (IoT) applications, BLE is identified as the least power consuming [28, 29]. A further motivation for using the CC2650 is the fact that it comes with a built-in 12-bit ADC; this further increases the system integration and relaxes the PCB footprint. A receiver is required to accept the data from our micro-instrument and forward it to the cloud application. Thus, functioning as a receiver, a CC2650 development board was connected to an internet-linked PC through the universal asynchronous receiver-transmitter (UART) protocol. However, any device that supports BLE and has internet connectivity may be used as the receiver.

**Fig. 4:**
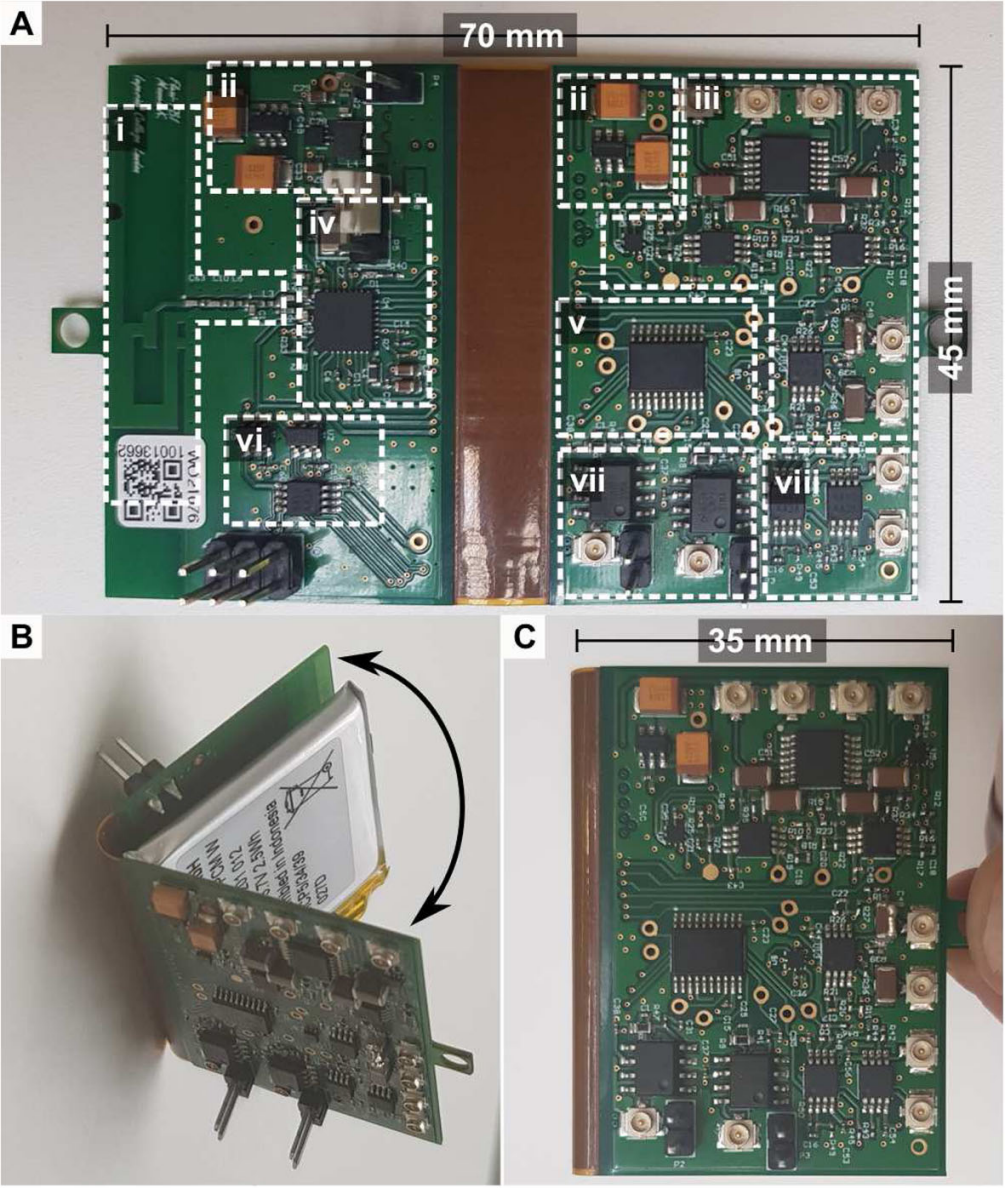
The manufactured flexible PCB. The mico-instrument: **a** The realisation of the design on a flexible four-layer PCB consisting of: **(i)** the printed planar inverted-F antenna and matching circuit, **(ii)** the power regulation block, **(iii)** the ECoG AFE with six channels, **(iv)** the CC2650 microcontroller, **(v)** the programmable gain amplifier stage with a built-in multiplexer - PGA117 **(vi)**, the programmable potentiostat, **(vii)** the dual-channel amperometry AFE and **(viii)** the dual-channel potentiometry AFE. **b** The folding of the device. **c** The folded device occupying half the original area

Figure 4a-i shows the antenna and matching network; a printed planar inverted-F antenna has been etched onto the copper layer. The figure presents the power regulation circuity (ii), the six-channel ECoG AFE (iii), the microcontroller with the integrated ADC and transceiver (iv), the PGA stage (v), the potentiostat (vi), the two-channel amperometry AFE (vii) and the two-channel potentiometry (viii). The architecture has been implemented on a four-layer flexible PCB. The board can be folded, which halves its size and allows stowing of the battery in between as shown in Fig. 4b, c. Finally, the device specifications are summarised in Table 1.

**Table 1:** Device specifications summary

Specification	ECoG AFE	Amperometry AFE	Potentiometry AFE
Number of Channels	6	2	2
Resolution	12 bits	12 bits	12 bits
Max Sampling Rate	1.25 ksps	312 sps	312 sps
Input Range	± 10 mV	± 100 mV	± 1.5 nA
Frequency Bandwidth	30 Hz	10 Hz	10 Hz
Input Referred Noise	9 nV/$\sqrt {Hz}$	0.02 pA/$\sqrt {Hz}$	9 nV/$\sqrt {Hz}$
Board Dimensions		3 × 4.5×1 cm^3^	
Board Weight		16 g	
Power Consumption		66 mW (3.3 V – 20 mA)	

### Software architecture

#### Embedded firmware

**Fig. 5:**
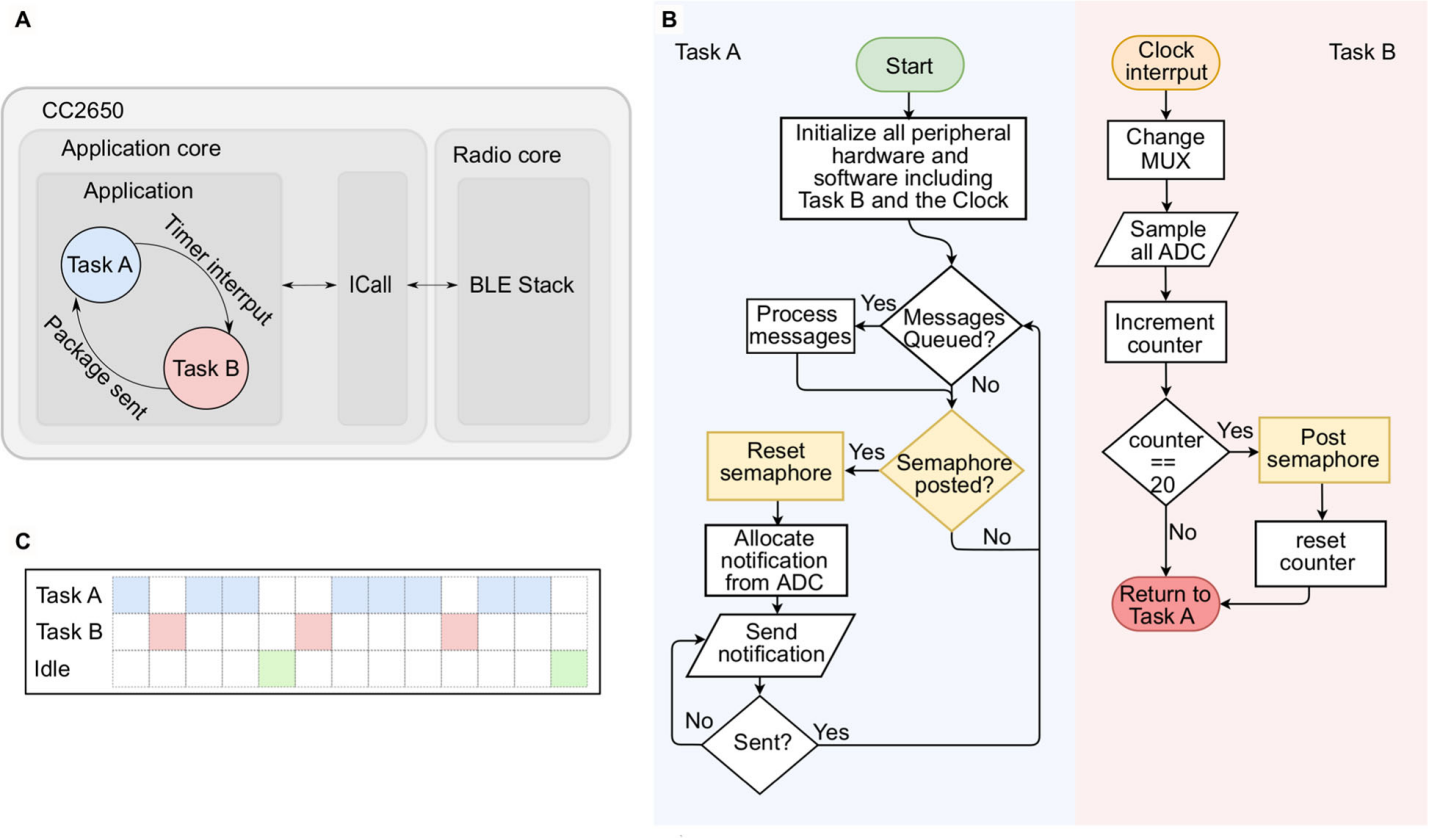
The embedded firmware. **a** A hardware perspective of the firmware running on the CC2650. The main application switches between the two tasks. The radio core runs the BLE stack and communicates with the application core via ICall. **b** The flowchart of the two tasks: Task A is responsible for device initialisation and higher level coordination. Task B is responsible for the uniform sampling of the different ECoG and chemical channels. **c** The time schedule of the CC2650, showing the time spent on Task A, Task B and instances when the controller is idle

The CC2650 is a dual-core micro-controller. It consists of a radio core (ARM Cortex-M0) responsible for BLE communication and data dispatch, and a main application core (ARM Cortex-M3) as shown in Fig. 5a. Synchronisation and data transfer between the two cores is facilitated by means of a messaging framework termed ICall. The CC2650 natively supports a real-time operating system (TI-RTOS), which greatly simplifies task scheduling and resource sharing between the different cores and tasks.

With regards to the firmware, there two main tasks running on the application core. The first task (Task A) is responsible for hardware initialisation, which includes starting and setting the operation parameters of the ADC, timers, clocks, ICall and the intra-board digital communication protocols (i.e. SPI, I2C). Even though the BLE operations are handled by the radio core, they need to be registered and defined in this task using ICall. Mostly important for this task is parsing the ready data, sending it out to the receiver and ensuring delivery. The general flow chart is shown in Fig. 5b.

The second task (Task B) samples input signals from all the channels in the ADC. Accurate, timely and uniform sampling is of paramount importance. To ensure that the first task load does not interfere with the sampling rate, this task is triggered by a high priority timer interrupt. More specifically, the interrupt callback is triggered at the start of every sampling period to sample all the six ECoG channels and one (out of the four) chemical channels selected by the multiplexer. The other chemical channels are sampled sequentially over the next three callbacks. Hence, the chemical channels are sampled at a quarter of the ECoG channels’ sampling rate; this is acceptable for this application lower speed requirements for sampling chemical biomarkers compared with ECoG. When the sampled data is ready, it is packed in a notification and then a special semaphore is posted permitting Task A to send the notification. The flowcharts and time schedule for the tasks are shown in Fig. 5b and c, respectively.

**Fig. 6:**
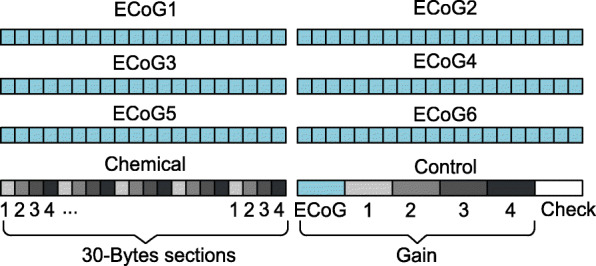
The notification data payload. The extended length BLE notification has a total length of 240 bytes. The notification is divided into eight blocks of 30 bits each. The first six blocks each contain 20 samples for each of the ECoG channels, while the chemical block contains 5 samples for each of the two potentiometric and two amperometric channels. The last block holds the gain value for each channel and a unique package identifier for connection error and loss detection

BLE protocol sends data as notifications, with each notification having a 7-byte protocol overhead. The effective notification length, excluding the overhead, is referred to as maximum transmission unit (MTU). Admittedly, BLE is not designed for high-throughput data streaming due to the big overhead and restricted notification length in Android and iOS [30]. However, by using a custom receiver and editing the BLE stack, data length extension (DLE) allows for expanding the length of the notification to 251 bytes (244 bytes MTU) [31]. This calls for a coarse-grained approach to the problem, where many samples can be bundled in one long notification reducing the overhead and maximising the throughput. Since the ADC data are 12 bits long, to fully exploit the notification size twenty samples for each channel are bundled into one notification, together with relevant information such as the gain values used in the ECoG, potentiometric and amperometric channels as shown in Fig. 6.

It is the receiver-side responsibility to unparse the notification back to their corresponding channels and to convert the raw ADC data to measured units using the gain information found in the least-significant block of the notification as shown in Fig. 6. These tasks were implemented with a Python daemon running on the host PC connected to the receiver. Finally, the processed data is sent to the cloud server.

#### Cloud application

**Fig. 7:**
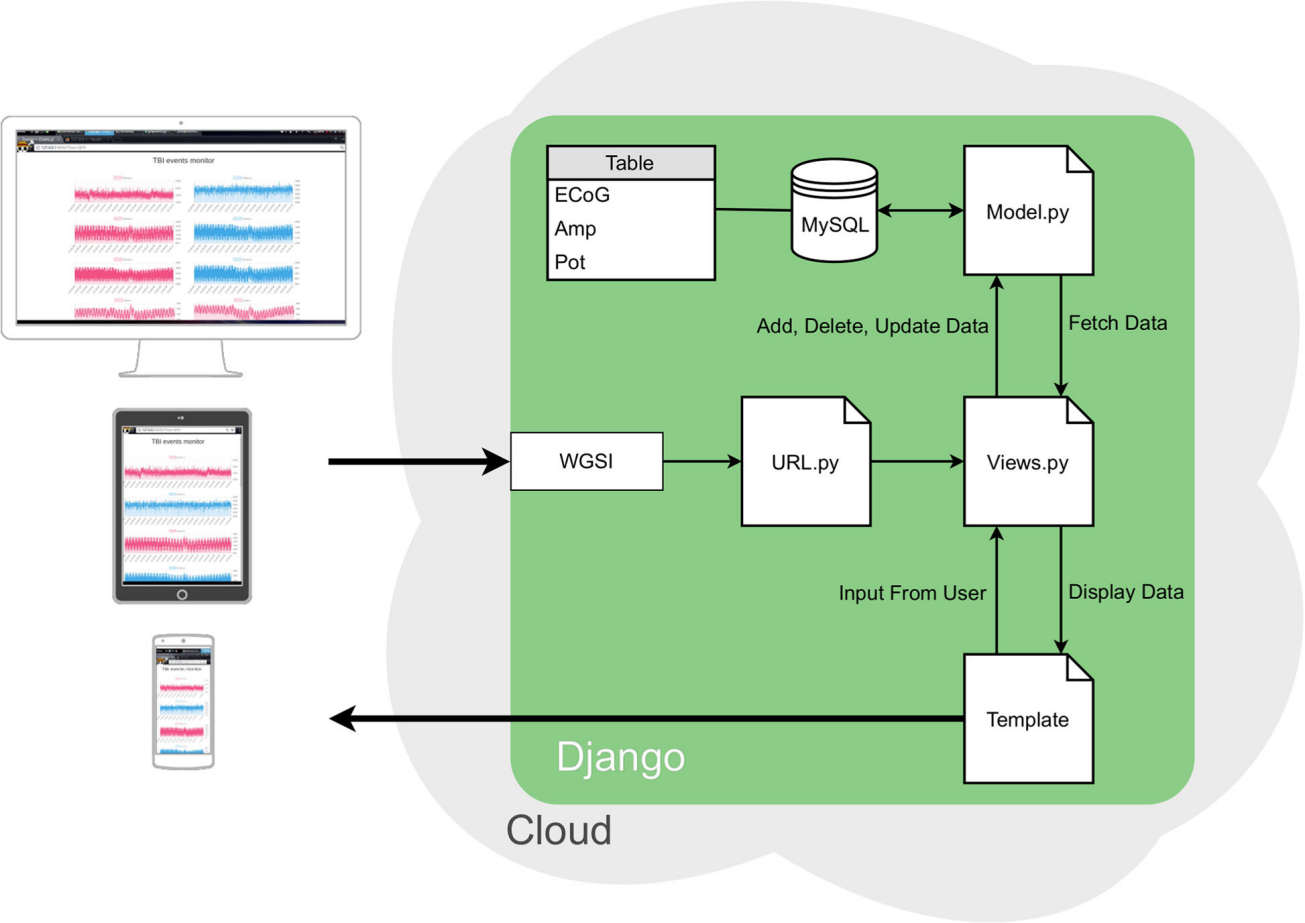
The cloud application architecture. The architecture is based on Django framework, whereby devices requests are translated by the WGSI and routed by *URL.py* to *the Views.py* which communicates with and controls *Models.py* and *template.py*. *Models.py* is responsible for interactions with the database where the ECoG, amperometric and potentiomteric recordings are stored. While *template.py* generates and returns the application visuals to the user’s device. The web application can run on different device sizes and platforms

The cloud application filters and analyses the received data as necessary, subsequently stores it into a database, and finally provides the tools and interface for data visualisation. The cloud service ecosystem is based on Python, a major language for data analysis [32]. This allows the development of robust and reliable event detection and personalised diagnostics algorithms. At the core of the ecosystem is Django framework, which is a high-level model-view-template (MVT) framework designed for rapid web development that offers high scalability and security [33]. The cloud server environment is shown in Fig. 7. The main motivation for choosing a web-based implementation for the software is to ensure cross-platform compatibility.

The web server gateway interface (WSGI) is the gateway between browser/client request and the cloud server response. URL requests are redirected to the controller (*Views.py*) through the *URL.py* script. *Views.py* is the core system controller, which takes the URL requests from the user and redirects the user to the correct template page. Additionally, it controls the receiving of data from the database (through *Model.py*) and parsing it into the appropriate format to be sent to the template when ready. The template is what appears on the internet browser of the device, as shown in Fig. 7. The template was programmed to render a real-time (refresh rate of 1 second) data animation and visualisation of each channel. Our template also offers users the ability to control how many samples (or the time window) are shown on the screen as well as allowing scrolling to past values. JavaScript (Chart.js library) has been used to develop the template.

The *Model.py* script implements Django’s object-relational mapping (ORM), it translates Python codes to database queries. For each table in the database a separate model is defined. Through these models, data in a database tables can be added, fetched, edited, and deleted. In our system, a MySQL database is used with one table made up of ten columns, each for a separate channel on the device. When all ADC channels are sampled at 1 ksps (250 sps for each chemical signals), and if a unique transactions is executed for storing each sample, there will be a 7,000 database transactions per second. Handling such number of transactions increases the server hardware requirements and price. However, since the data does not arrive at uniform intervals, because a coarse-grain approach is used in the transmitter; it is better to handle the database transactions in coarse-grained fashion as well: data is buffered, bundled and inserted to the database using one bulk transaction instead of separate transactions for each sampling, reducing the total overhead and execution time.

## Results

In this section, the performance of the system is examined in two ways: Firstly, the functionality of the different blocks on the instrument are tested in isolation. Secondly, the complete system stack, inclusive of the instrument and cloud application, is tested by measuring neuroelectrical (ECoG/SD) and neurochemical signals (glucose, lactate, potassium).

### Block-Level performance

#### Voltage measurements

**Fig. 8:**
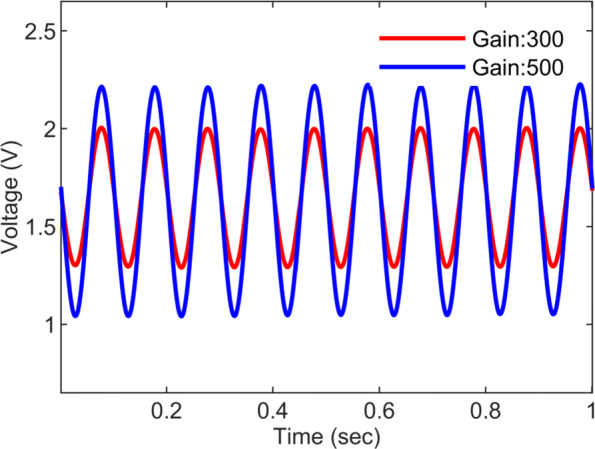
Testing the ECoG AFE. The ECoG AFE response to a 10 Hz, 2 mV _*pp*_ input signal when the gain is set to × 300 and × 500

The ECoG AFE has been tested with a 10 Hz, 2 mV _*pp*_ input signal while first setting the gain to × 300, and then to × 500. The input signal was produced with a function generator (GW Instek AFG-2125). The signals at the AFE output, shown in Fig. 8 for both gain values, were recorded using a PowerLab data acquisition (DAQ) device. The potentiometric AFE has a similar design to the ECoG AFE, the only difference being the gain value set by the PGA.

#### Current measurements

**Fig. 9:**
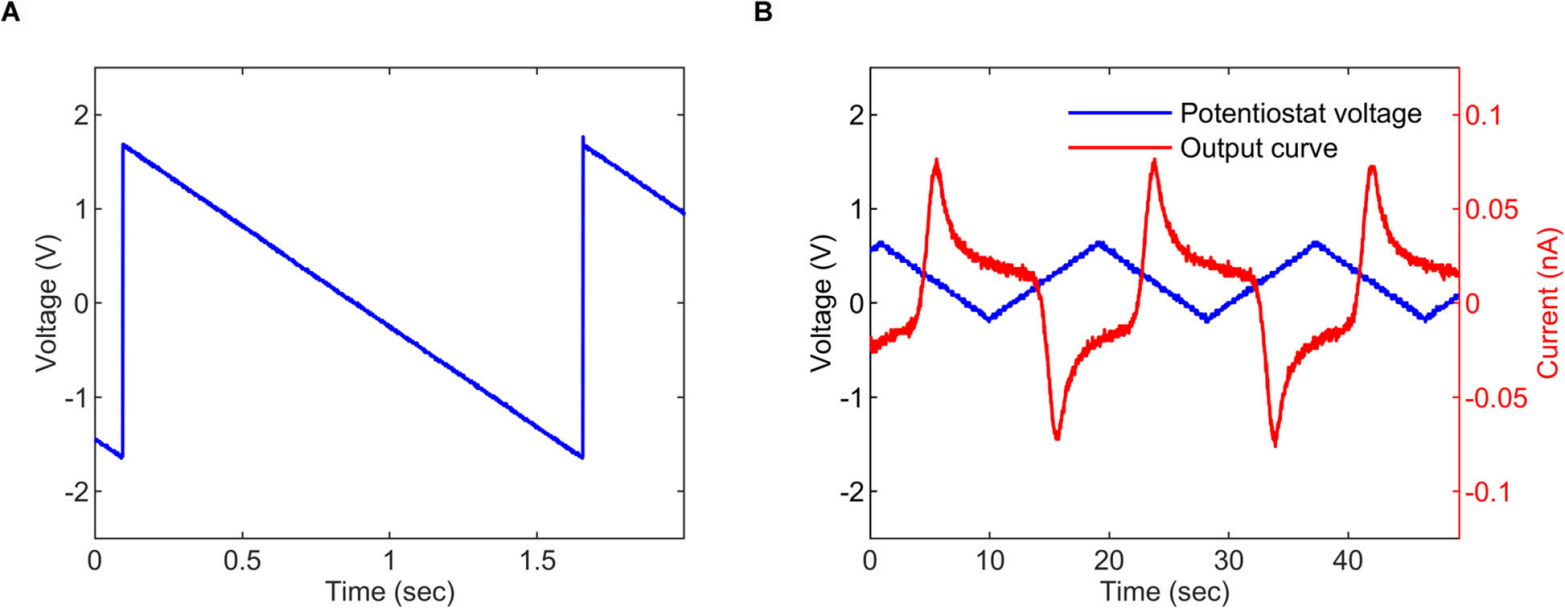
Testing the potentiostat and running cyclic voltammetry. **a**The potentiostat-generated voltage sweep starting from 1.65 V down to -1.65 V, the voltage is measured on the working electrode (WE) with respect to the reference electrode (RE). **b** Cyclic voltammery output curve for a 30 *μ*M ferro-ferricyanide solution. The potentiostat is periodically swept between 0.6 V and -0.2 V with a scan rate of 80 mV/s

To run amperometry measurements, the potentiostat must be able to generate an accurate range of voltage values to apply to the working electrode to run the reaction. This was verified by programming the potentiostat to produce a ramp function starting from 1.65 V down to -1.65 V. The output was acquired with a PowerLab DAQ and plotted as shown in Fig. 9a. Next, to further prove the flexibility of potentiostat its robustness in generating waveforms, cyclic voltammery was run in a 30 *μ*M ferro-ferricyanide solution using a carbon electrode (WE), Ag |AgCl electrode (RE) and Pt wire (CE). The resultant measurement after conversion from current to voltage and amplification by the amperometric AFE is shown in Fig. 9b.

**Fig. 10:**
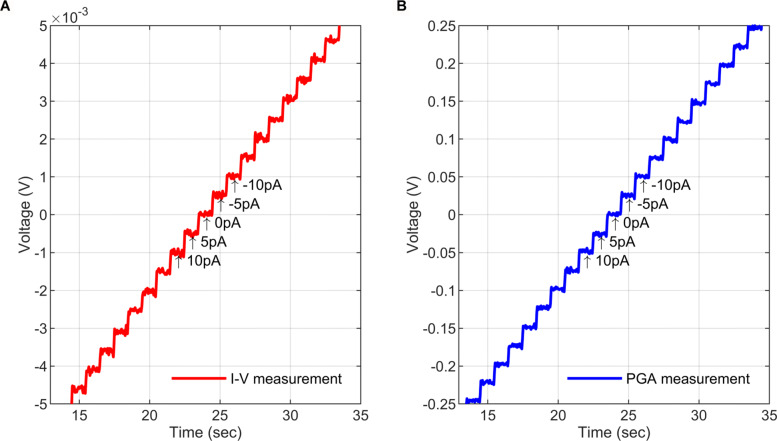
Resolution of the amperometric AFE. A low-noise source-generated current staircase sweep (-50 pA to +50 pA with steps of 5 pA) was fed to the amperometric AFE. The voltage measured is shown at: **a** The I-V stage output. **b** The output of the programmable gain amplifier (PGA) with the gain set to × 50

To examine the resolution of the current-to-voltage (I-V) stage, an ultra-low-noise precision current generator was used (Keithley6221) to produce a staircase sweep from -0.1 nA to +0.1 nA with a 5 pA step. Figure 10a reports the I-V measurement, which shows that the I-V stage maintains the 5pA steps accurately after trainsamplification with 100 mV/nA gain. In addition, the PGA stage gain was set to × 50, and the 5 pA steps were observed at the output of the PGA after the additional amplification, as shown in Fig. 10b. The result confirms that the AFE can accurately measure low-frequency currents with amplitudes as small as a few pA with little noise introduced.

#### Multiplexing and reconstruction

**Fig. 11:**
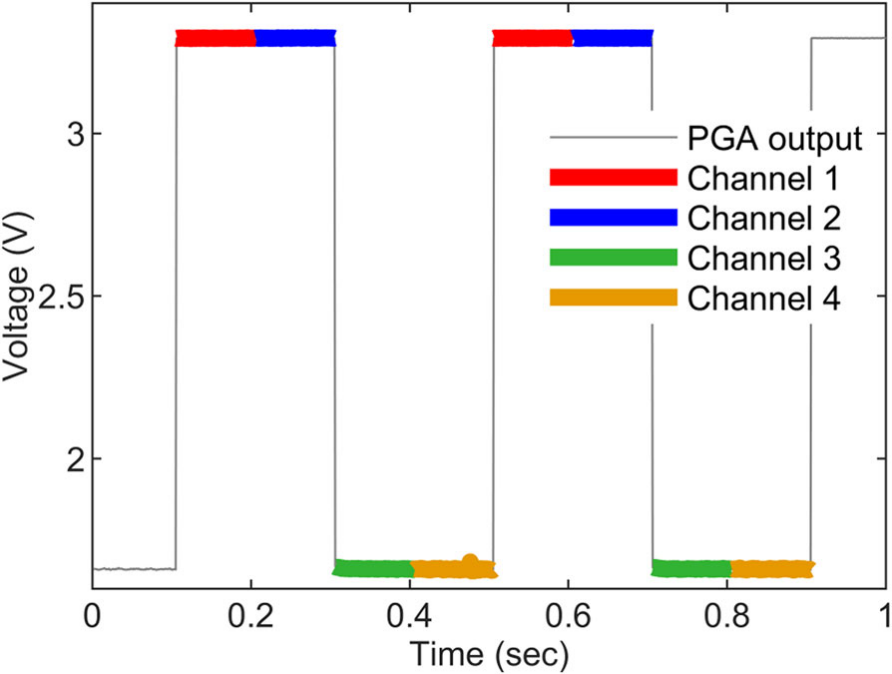
Multiplexing and reconstruction of the neurochemical inputs. The operation of the PGA’s integrated multiplexer and the decomposition algorithm: The PGA’s multiplexer combines the four chemical channels into one output line, shown as the grey trace. The decomposition algorithm reconstructs the original inputs back into their correct time slots, this is illustrated with the coloured traces

As previously explained, the integrated multiplexer block plays an important role towards the instrument miniaturisation, power consumption reduction and performance optimisation by combining all the slow varying chemical signals into one channel. This was examined by setting two channels at 3.3 V and the other two channels to 0 V, then the output at the PGA117 was recorded using a PowerLab DAQ as illustrated by the grey trace in Fig. 11. On the server side, The channels were algorithmically decomposed into their correct time slots accurately. Each separated channel output is colour-coded and overlaid on the grey trace in Fig. 11.

#### Wireless BLE communication

**Fig. 12:**
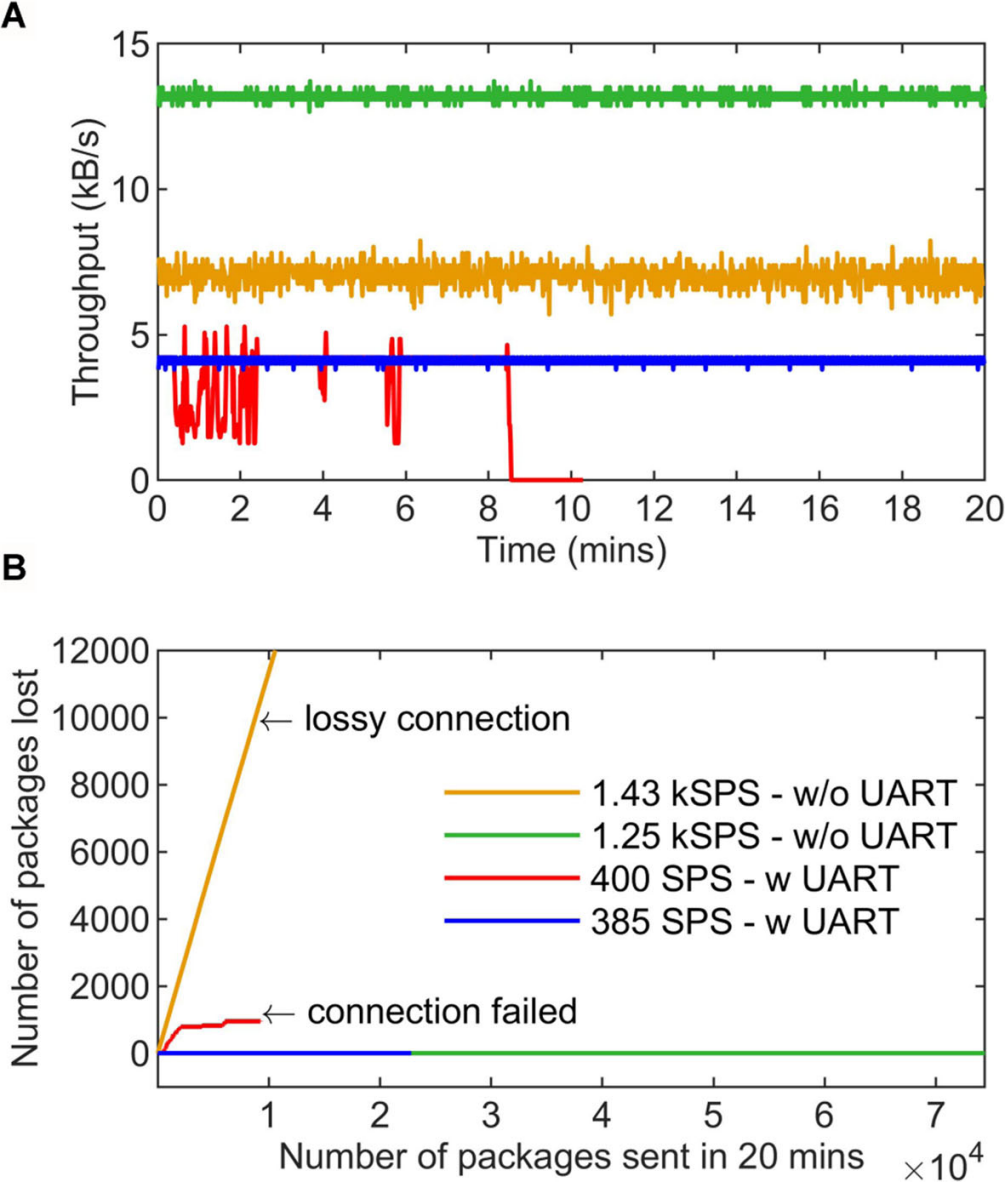
Characterisations of wirless link quality and sampling rate. **a** Different throughput values for sampling rates of 1.43 ksps/channel without UART, 1.25 ksps/channel without UART, 400 sps/channel with UART and 385 sps/channel with UART. Measurements were taken for a total of seven channels during a period of 20 minutes. **b** Connection quality is measured as the number of packages lost for the different sampling rates. When using UART the connection is stable for 385 sps/channel but fails for 400 sps/channel and higher; Otherwise the connection is stable for 1.25 ksps/channel but starts becoming lossy at 1.43 ksps/channel

To assess the BLE wireless channel performance, throughput and loss have been calculated for several sampling rates ranging from a few hundred to a few thousand hertz. Measurements took place with the receiver placed one metre away from instrument. Two sets of measurements were taken: with UART at 115200 baud rate and without UART. When UART was used to connect the receiver with a PC running the Python daemon, a maximum of 385 sps/channel was achieved as shown in Fig. 12a. Using 400 sps/channel led to connection failure due to the receiver’s processor running out of resources to successfully receive the data from the instrument and send it with UART to the cloud-connected PC. Without using UART, the setup achieved a maximum throughput of 1.25 ksps/channel as depicted in Fig. 12a. At around 1.43 ksps/channel the connection became lossy as the transmitter was running out of resources this time. This is clearly illustrated in Fig. 12b where for 1.43 ksps/channel a significant number of packages are lost; for 400 sps/channel (UART) the connection drops; and for 1.25 ksps/channel and 385 sps/channel (UART) the connection is stable. It is concluded that UART introduces a bottleneck reducing the maximum throughput from 1.25 ksps/channel to 385 sps/channel.

### System-Level performance

#### Biopotential measurements

**Fig. 13:**
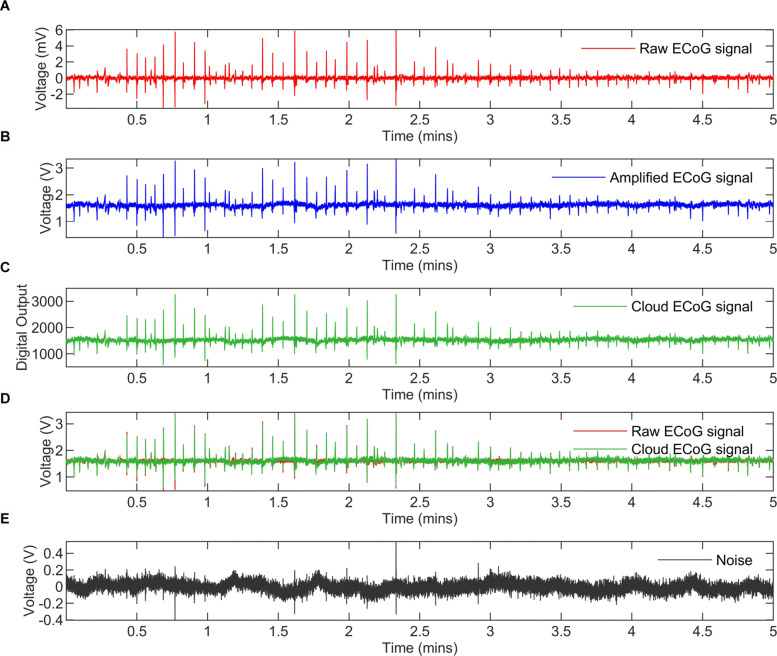
ECoG signals from measurement to cloud. **a** Raw ECoG signal recorded from a patient experiencing spreading depolarisation (SDs) and played back to scale with a function generator and an 20 dB attenuator **b** The ECoG signal after amplification measured at the output of the ECoG AFE **c** The digitised signal at the cloud server measured after analog-to-digital conversion and wireless transmission. **d** The reconstructed ECoG signal in the cloud and the scaled raw ECoG signal **e** The noise introduced from interference, amplification, filtering, analog-to-digital conversion and wireless transmission

ECoG recording from a patient experiencing SDs was played back using an arbitrary waveform generator (Agilent 33220A) and a 20 dB attenuator. The generated signal, depicted in Fig. 13a, matched the magnitude of the original clinical recording. The signal’s trend clearly shows the suppression of the low-frequency cortical activities at around 3 mins, which is indicative of the occurrence of SDs. The system assessment criterion is that the amplification, filtering, transmission and reconstruction of the ECoG AFE must have an accuracy high enough to preserve the suppression trend.

With the ECoG AFE gain set to × 300 and the sampling rate to 250 sps/channel, the ECoG signal was measured at the output of the AFE (Fig. 13b), and at the server (cloud) side after transmission and digitisation (Fig. 13c). Finally, the reconstructed signal is shown and compared with the original raw ECoG in Fig. 13d. Clearly, the system still preserves the profile necessary to identify the SD. Signal-to-noise ratio (SNR) was calculated, using equation 1, to quantify the noise/difference between the raw and server ECoG signals:

1 $$\begin{array}{@{}rcl@{}} SNR &=& 20 \log{\left[\frac{V_{signal}}{V_{noise}}\right]} \end{array} $$

The noise is plotted in Fig. 13e and the SNR is found to be 29.07 dB. Additionally, the digitisation and transmission noise factor (NF), equal to 0.26 dB, was calculated by subtracting the SNR at the server (29.07 dB) from the SNR after amplification (29.33 dB).

#### Chemical biomarkers measurements

**Fig. 14:**
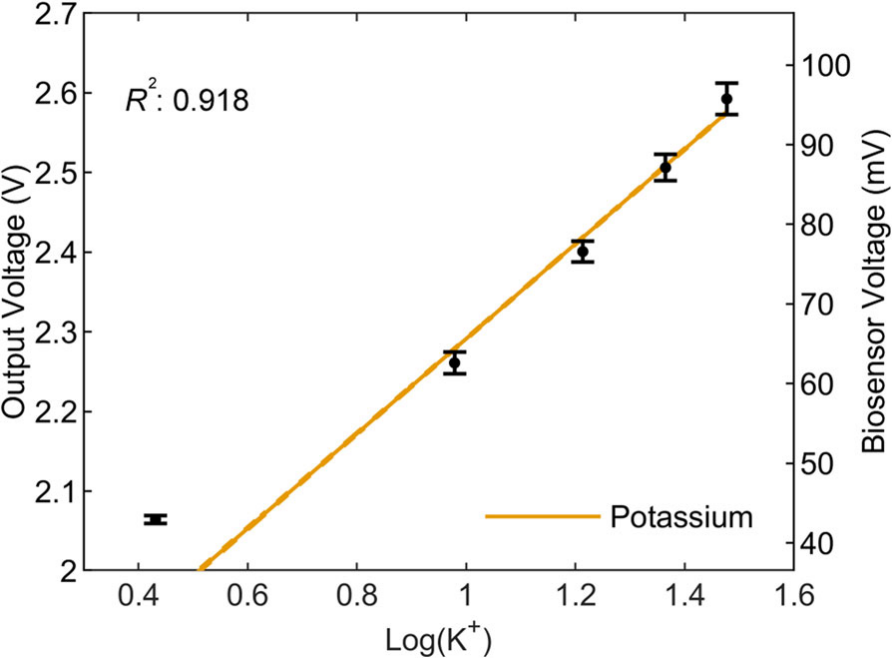
Potassium measurements. The generated logarithmic working curve for potassium measurements of concentrations between 2.7 mM to 30 mM in a physiological buffer. The curve shows an R^2^ of 0.918. n= 2000 samples for each concentration point. Error bars show mean and standard deviation

To examine the performance of the system with chemical potentiometric measurements, five concentrations from 2.7 mM to 30 mM of potassium in artificial cerebrospinal fluid (aCSF) were pumped through a PDMS microfluidic chip. A potassium ion selective electrode (ISE) was placed in the chip and connected to the board. The potentiometric AFE gain was at × 1, the PGA gain was set to × 10 and the sampling rate to 62.5 sps/channel. The digital data was exported from the cloud server after transmission, it was then re-converted to analog voltage values and filtered with a 10 Hz cut-off, 17th-order Chebyshev II low-pass filter. Since the output voltage and concentration show a logarithmic correlation, logarithmic regression was used to construct the working curve as shown in Fig. 14. The line has an R^2^ value of 0.918% and a slope of 59.5 mV/decade. Noticeably, the deviation at low potassium concentration is due to interference from competing sodium ions in solution.

Furthermore, simultaneous amperometric measurements of glucose and lactate were carried out to characterise and verify the system performance. An autocalibration board consisting of LabSmith programmable syringe pumps and valves [34] was used to generate steps of different concentrations from 0 to 1 mM with steps of 0.25 mM for both biomarkers. The potentiostat was used to set the potential at the WE to +0.7V above the RE. The sampling rate was kept at 62.5 sps/channel, and the PGA gain was set to × 5. The biosensors were placed in a 3D printed microfluidic chip and connected to the instrument via three electrodes (WE, RE, CE) [35, 36].

**Fig. 15:**
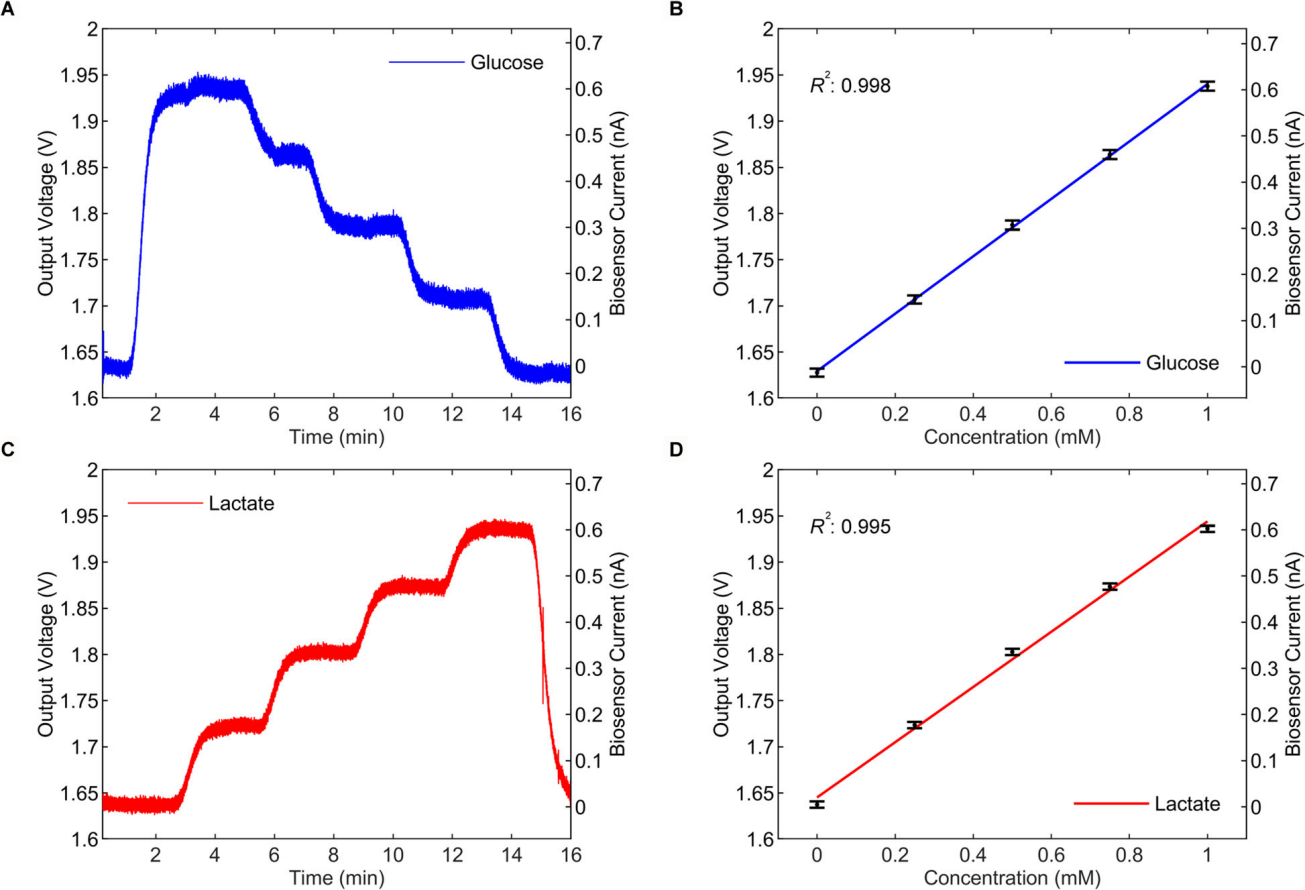
Glucose and lactate measurements. **a** Glucose measurements for a 5-point calibration from 0 to 1 mM with steps of 0.25 mM in a physiological buffer (T1), the total transimpedance gain is set to 5 × 100 mV/nA. **b** The generated working curve for glucose has an R^2^ of 0.998 and an LOD of 0.85 *μ*M. n= 2000 samples for each concentration point. Error bars show mean and standard deviation **c** Similar lactate measurements with concentrations from 0 to 1 mM and a total gain of 5 × 100 mV/nA **d** The generated working curve for lactate shows an R^2^ of 0.995 and an LOD of 1.3 *μ*M. n= 2000 samples for each concentration point. Error bars show mean and standard deviation

To measure glucose, a biosensor made of an enzyme-coated (glucose oxidase - GOx) 50 *μ*m Pt WE, a Ag |AgCl 50 *μ*m RE and a 27 gauge needle as the CE, was used [35, 36]. The measured results, reported in Fig. 15a, were extracted from the cloud server and filtered with the same digital filter used for processing the potassium measurements. Using linear regression the glucose working curve was generated as in Fig. 15b with an R^2^ of 0.998 and LOD of 0.85 *μ*M. Similarly, lactate was measured with a lactate oxidase (LOx) 50 *μ*m Pt WE [35–37]. The extracted lactate data is presented in Fig. 15c and the lactate working curve was generated with an R^2^ of 0.995 and LOD of 1.3 *μ*M as illustrated in Fig. 15d.

## Discussion

This technology represents a major step forward in monitoring TBI patients both for scientific research and to guide patient care. In order to understand the complexity of damage caused by TBI it is critical to be able to follow patients through their entire clinical journey. However, our current bedside instrument is only suitable for monitoring patients during the time in which they are placed in a drug-induced coma to lessen the metabolic burden on the injured brain. Migrating to wireless instrumentation as described here would allow baseline monitoring to be established early in the patient’s clinical journey, in contrast to current protocol in which monitoring only begins once the patient is set up in the ITU (up to 4-5 h after hospital admission). This would provide information about the evolution of the injury in this critical time period. Moreover, current protocol requires that monitoring be stopped as soon as sedation is lifted as patients are often disorientated and confused due to a combination of prolonged sedation and their injury; as the current monitoring setup requires wires and tubing to reach the bedside instrument, this makes such monitoring impractical for awake patients. The portable instrumentation presented here would solve this issue and allow TBI patients to be monitored even when awake and still at risk of spreading depolarisations.

There is a growing consensus that continuous multimodal monitoring is critical for TBI patients [10, 38–40]. Brain neurochemical signals are subtle and complex and as such high-quality time-aligned clinical data are needed to identify changes, particularly in a high-noise clinical environment. This would allow clinicians to control and optimise patient treatment and rehabilitation on an individual basis. The high-performance instrumentation presented here is a significant step towards achieving these goals.

The device complies with the IEC 60601 standard. It is battery operated and does not provide a direct line between the patient and the mains electricity. In addition, the chemical sensors are not implanted directly in the brain tissue, instead a clinically-approved microdialysis probe samples the brain fluid into the microfluidic chip where the sensors are placed via a length of fine bore tubing. With regards to data security and patient privacy, all data collected is anonymised at source as per GDPR guidelines.

***Limitations***

Several limitations to the instrument presented in this study need to be considered; the instrument is only suitable for the measurement of low frequency ECoG signals in the range of ≤ 30 Hz. Moreover, all filters in the instruments are first order, hence, additional digital filters are required at the server side for sufficient noise cancellation.

Also, as pointed out in the results section, the receiver design is a limiting factor with regards to the sampling rate and data throughput. Using UART requires significant resources from the CC2650, which can lead to the BLE connection with the mico-instrument being compromised. However, this can be rectified either by replacing the CC2650 in the receiver with a more powerful controller, or by using a different protocol such as a parallel bus. However, both of these solutions would increase the complexity and cost of the receiver. Nevertheless, for as far as TBI is concerned, the current receiver design supports sufficient sampling rates, i.e. 385 sps/channel.

Finally, some potassium ISEs had a voltage offset that varies from one batch to another. The offset which can reach up to 200 mV can lead to the input exceeding the designed input range of the AFE. To address this, a digital-to-analog converter (DAC) might be incorporated in the potentiometric AFE to cancel out the offset by dynamically adjusting the RE potential.

***Future work***

Presently, sensor calibration is carried out using an independent remote calibration setup. However, as our micro-instrument is able to generate different waveforms, run cyclic voltammetry and calculate working curves, it could be used to carry out and control calibrations. This feature would result in better utilisation of hardware and a reduction in the system complexity by enabling an all-in-one solution. Further work is required to establish this.

In future investigations, it might be possible to exploit the cloud infrastructure described in this article for the development and testing of data mining and machine learning algorithms targeted for SD event detection, diagnostics and condition risk assessment, prognosis of TBI-related complications, rehabilitation and personalised medicine. The same instrument is fit for use in rapid hormonal testing (such as cortisol), which is important for athletes and patients with hormonal disorders. Other possible applications are the monitoring of stroke and epilepsy.

On a final note, the combination of the flexible PCB presented in this account and the microfluidic device presented in [36] leads to the realisation of the complete system as shown in Fig. 1. Before an early feasibility clinical trial could be initiated, more work is needed to integrate all hardware components into a single wearable device package and to acquire the necessary ethical approval.

## Conclusion

This project was undertaken to design a system for wireless real-time monitoring of TBI patients. It trades off a degree of performance for reduced cost and size. The paper describes the system’s hardware and evaluates its performance in measuring biopotentials and neurochemical biomarkers. It also proposes a cloud server infrastructure for data storage and visualisation.

The mico-instrument ECoG AFE has been confirmed to support two gain values × 300 and × 500, and to accurately measure clinical ECoG recordings with a rich quality of features. By comparing the raw recording with the acquired ECoG signal at the server after amplification, digitisation and transmission, it was validated that the instrument has high accuracy. This enables the detection of the suppression of low-frequency brain activity associated with SDs. Furthermore, the noise induced by the instrument was analysed and estimated by calculating the SNR (29.07 dB) and the NF (0.26 dB).

With regard to chemical measurements, the ability of the device to support several electrochemical techniques, including potentiometry, amperometry and cyclic voltammetry, has been proven. The amperometric AFE section of the board was rigorously tested with low-noise bipolar currents establishing its high resolution of less than 5 pA. It was also verified that by exploiting the slow dynamics of the chemical biomarkers, it is possible to allocate only one ADC channel for all the chemical channels by means of an integrated multiplexer and programmable gain amplifier stage. Using such an approach led to cost and size benefits. Additionally, the same PGA stage can be programmed with several gains from × 1 up to × 200 to measure various chemical biomarkers with different sensitivity ranges. Finally, both the potentiometric and amperometric AFEs were used to measure biologically relevant concentrations of potassium, glucose and lactate with the use of micofluidic chips and biosensors.

The study went on to analyse the capabilities and limitations of the BLE protocol and identified UART as the communication bottleneck capping the sampling rate at 385 sps/channel. However, the system can achieve 1.25 ksps/channel if other protocols or an alternative processor were used in the receiver.

In conclusion, we presented a complete system tailored to TBI neuroelectrochemical monitoring, that provides aggressive cost and size advantages without compromising measurement accuracy. For future studies, we see the instrumentation expanded to monitoring of other conditions such as stroke and hormonal disorders, and the cloud server functionalities extended for cortical event detection, diagnosis and personalised medicine applications through the exploitation of machine learning techniques.

## Data Availability

Data sharing not applicable to this article as no datasets were generated or analysed during the current study.

## Abbreviations

TBI: Traumatic Brain Injury
BLE: Bluetooth Low Energy
SD: Spreading Depolarisation
ICU: Intensive Care Unit
HDU: High Dependency Unit
ECoG: Electrocorticography
L/P: Lactate/Pyruvate
co-MD: Continuous on-line Microdialysis
AFE: Analog Front-End
ADC: Analog-to-Digital Converter
PGA: Programmable Gain Amplifier
WE: Working Electrode
RE: Reference Electrode
CE: Counter Electrode
I-V: Current-to-Voltage
TIA: Transimpedance Amplifier
ANT: Adaptive Network Topology
IoT: Internet of Things
UART: Universal Asynchronous receiver-Transmitter
MTU: Maximum Transmission Unit
DLE: Data Length Extension
MVT: Model-View-Template
WSGI: Web Server Gateway Interface
ORM: Object-Relational Mapping
